# The Single or Second Dose

**Published:** 1885-03

**Authors:** Rollin R. Gregg

**Affiliations:** Buffalo


### THE SINGLE OR SECOND DOSE.

EDITOR HOMŒOPATHIC PHYSICIAN:—The following quotation from Hahnemann in your last October's issue, ought to be put into letters of gold, or, better still, into letters of life, if that were possible, to startle men into a recognition of the truth.

"Let no one come, then, and say a remedy has been given in an appropriate case; in the strongest doses and not too seldom either, but every two or three hours, and *yet* the patient is dead. Nay, I reply, from full conviction, *for that very reason* he is dead, and thou hast killed him. Hadst thou given him a single dose of the smallest part of a drop of the twenty-fifth or thirtieth dilution (in rare cases a second dose, repeated on the third or fourth day), then had the patient been saved *certainly*, and with much less trouble."

I wish these words could be burned into the hearts and consciences of men so deeply as to leave a scar that would stand as a constant protest and warning against the frequent repetition of doses, excepting in "rare cases."

You say these words of Hahnemann were "from a note to the proving of Hyoscyamus." Let me give you a case in point. Fifteen or eighteen years ago I treated one of the worst cases of typhoid fever I ever saw—a pure Hyoscyamus case. The patient was a young lady in her seventeenth or eighteenth year, and of previously very delicate health all her life. The great characteristic feature of her case was a delirium, in which she was almost constantly talking when awake of going home, going away, etc., and almost as constantly making efforts to get out of bed. The only way anything could be done with her was to put on her skirts, stockings, and shoes, a sacque and sometimes a bonnet, and allow her to sit bolstered up upon the edge of the bed with her feet in a chair. This would partially pacify her, and she would sit for an hour or more in that way until completely exhausted; then she could be gotten into bed again for a time, a few hours at most, when the same process had to be repeated; and so on day after day for a week or more. Other symptoms were equally serious, and the case had in all its bearings such an ominous outlook that not one of many intelligent friends had the slightest hope of her recovery. In this want of hope I fully shared. Among the other bad symptoms, a firm cast of sordes formed upon the whole roof of her mouth, completely covering it from the teeth back to the palatine arches, where it was at least half an inch in thickness and interfered very seriously with deglutition. No ordinary efforts to dislodge this mass made the slightest impression upon it, so it was allowed to remain.

At the beginning of this case, and for a week or more, until the Hyoscyamus symptoms developed into such prominence, I gave Bryonia, Rhus tox., Belladonna, and one or two other remedies, a few doses each, but, of course, with no beneficial effect. Finally there could be no longer a doubt as to the remedy indicated, and one dose of Hyoscyamus $^{1000}$ (Jenichen) was given and results awaited. There was a decided amelioration in a day or two of the worst symptoms, from that dose; still at times there was yet such great severity in those symptoms as would have forced many to have given repeated doses. But I held on resolutely, seeing no hope in the case excepting through the greatest caution and no mistakes being made. After four or five days, and from an apparent increase in the symptoms, that held on longer without intervals of relief, a second dose of the same remedy and potency was given. Following this, a more decided improvement was manifested, the patient quieted down and could be kept in bed, and from that on I had little further anxiety in the case. Within a few days after that second dose, the cast of sordes on the roof of the mouth loosened and was detached, but so large was it that the patient could not expel it. It came near strangling her to death when it became detached, and the nurse had to seize it and pull it out of her mouth. After that, convalescence was quite rapidly established, and no more medicine was required excepting a single dose each of Pulsatilla and Nux vomica for some symptoms that remained and were somewhat annoying, but in no way of serious import. Nor was this all. The young lady, who, as I have said, had been very delicate all her life, became strong and robust after her recovery from the fever without more medication; and, moreover, has never been sick since, excepting a very severe cough from a neglected cold a year ago last spring, but which one dose of Phosphorus cured completely in a week or ten days after she had allowed it to run three or four weeks.

Now, I have not the slightest doubt that this patient would have speedily died under repeated doses of Hyoscyamus. It was one of the worst cases of typhoid fever I ever saw, whether the patient lived or died. However, I know that severe cases are sometimes saved by repeated doses of medicine. But how do such get up? Almost invariably they are left with some chronic disease of a more or less serious nature, from which many of them ultimately die. A case in point just occurs to me.

Over thirty years ago my old preceptor was called to a young lady who had been violently ill of bilious fever ten days or a fortnight, and was given up to die by her attending allopathic physician. Some of her more prominent symptoms were attempts to get up, expressions of a desire to go home, picking at the bed-clothes, etc. The doctor gave her Hyoscyamus$^{6}$ in alternation with some other remedy every two hours. He had the great satisfaction of raising his patient from her sick-bed in two or three weeks, but she then went into consumption and died in a year or so after of that disease.

I have seen not a few similar results, and from a great deal of observation in such matters, during these more than thirty years, I have little hesitation in saying that had no more than two doses in all of Hyoscyamus been given in that case, with a dose or two of any other remedy that might have been called for later, that patient would have been restored without lung disease having been developed out of it, and been left as well or better than before taken down with the fever. A general application of these facts I would make in all acute diseases. Therefore, in conclusion, let Hahnemann's words be burned into the consciences of men, until they will heed them and *save* their patients, instead of killing them with too much medicine.

ROLLIN R. GREGG, M. D.

BUFFALO, January, 1885.

P. S.—An after thought comes up. If Hahnemann's warning and my experience in the treatment of most other violent diseases can be relied upon and extended to cholera, no more than two doses of any one medicine ought to, or can with entire safety, be given in any attack even of that disease, without a delay of twenty-four or more hours between the second and third doses of it, and a dose of one or more other remedies, if called for, between the second and third doses of the first remedy, if the third dose of the latter is absolutely required. Indeed, one of the most violent cases of disease I ever treated, and attended by as violent suffering as I ever saw, was a case of cholera. The attack had run on into the third day, rice-water discharges were pouring in floods at short intervals from both stomach and rectum, and such cramps and outcries from them as I never saw or heard before or since. The muscles of the abdomen, thighs, and calves would paroxysmally gather into knots as large as my fist, and not a rag of clothes could be kept on the patient to cover her nakedness in these paroxysms. She had torn her night-dress entirely from her before I was called and would not allow another to be put on. Well, this case was *wholly* relieved, discharges, cramps, and all, in six or eight hours by two doses of Cuprum met.$^{4000}$, at three or four hours interval, and convalescence thereby fully established without more medicine.

